# Multimer Detection System: A Universal Assay System for Differentiating Protein Oligomers from Monomers

**DOI:** 10.3390/ijms26031199

**Published:** 2025-01-30

**Authors:** Angelo Moscoso Jamerlan, Kyu Hwan Shim, Niti Sharma, Seong Soo A. An

**Affiliations:** Department of Bionano Technology, Gachon Medical Research Institute, Gachon University, Seongnam-si 13120, Republic of Korea; angelo@gachon.ac.kr (A.M.J.); smuller0305@gmail.com (K.H.S.)

**Keywords:** protein aggregates, oligomerization, neurodegenerative disease, multimer detection system

## Abstract

Depositions of protein aggregates are typical pathological hallmarks of various neurodegenerative diseases (NDs). For example, amyloid-beta (Aβ) and tau aggregates are present in the brain and plasma of patients with Alzheimer’s disease (AD); α-synuclein in Parkinson’s disease (PD), dementia with Lewy bodies (DLB), and multiple system atrophy (MSA); mutant huntingtin protein (Htt) in Huntington’s disease (HD); and DNA-binding protein 43 kD (TDP-43) in amyotrophic lateral sclerosis (ALS), frontotemporal dementia (FTD), and limbic-predominant age-related TDP-43 encephalopathy (LATE). The same misfolded proteins can be present in multiple diseases in the form of mixed proteinopathies. Since there is no cure for all these diseases, understanding the mechanisms of protein aggregation becomes imperative in modern medicine, especially for developing diagnostics and therapeutics. A Multimer Detection System (MDS) was designed to distinguish and quantify the multimeric/oligomeric forms from the monomeric form of aggregated proteins. As the unique epitope of the monomer is already occupied by capturing or detecting antibodies, the aggregated proteins with multiple epitopes would be accessible to both capturing and detecting antibodies simultaneously, and signals will be generated from the oligomers rather than the monomers. Hence, MDS could present a simple solution for measuring various conformations of aggregated proteins with high sensitivity and specificity, which may help to explore diagnostic and treatment strategies for developing anti-aggregation therapeutics.

## 1. Introduction

Proteins serve as fundamental mechanical, enzymatic, and structural components of living organisms. They are composed of one or more polypeptides, which are linear chains of amino acid residues linked by peptide bonds. Protein biosynthesis occurs through translation, wherein ribosomes decode messenger RNA (mRNA) sequences to assemble amino acids in a specific order. While the protein’s primary sequence primarily dictates its features by genomic DNA, secondary structures also play a crucial role in shaping and determining the protein’s functions from its amino acid sequence. Among the protein structures, aggregations and the excessive formation of β-sheets are associated with various neurodegenerative disorders (NDs), like Alzheimer’s disease (AD), Parkinson’s disease (PD), prion diseases, triplet repeat diseases, and dementia with Lewy bodies (DLB). The proteins associated with diseases, like amyloid-beta (Aβ) protein in AD, prion protein (PrP) in prion diseases, polyglutamine in triplet repeat disease, and α-synuclein in DLB, are consistent across different diseases [1]. Despite this, all the amyloid-forming proteins produce amyloids that have β-sheet structures, which are toxic. As a result, a new idea known as “conformational disease” was introduced, indicating that the shape of a protein plays a crucial role in its harmful effects and the subsequent onset of a disease [2].

Protein oligomerization is the association of two or more protein molecules or monomers to form a larger complex, an oligomer. This association is mainly driven by irreversible covalent bonds or through reversible associations mediated by electrostatic and hydrophobic interactions or hydrogen bonds. Oligomeric interfaces frequently exhibit considerable electrostatic and shape complementarity, leading to interaction specificity [3]. The oligomerization of proteins may have been evolutionarily valuable, as it would allow the establishment of large complex structures for higher biological functions and allosteric regulations, where enzymatic activities or original functions were inhibited through the binding of an inhibitor to a region outside the active site of the enzyme or altered structures [4]. Secondly, a shorter protein sequence in an oligomerized protein is more likely to have an error-free transcript. Several cellular systems or cell stability are maintained through proteostasis, such as the ubiquitin–proteasome system (UPS), chaperones, chaperone-mediated autophagy, and macro autophagy [5]. Neurons require special care to maintain proteostasis due to their complex structures, extended lifespan, and inability to reduce protein aggregates through cell division. The UPS plays a vital role in the proper operation of neuronal synapses, including the turnover of synaptic proteins, plasticity, and the formation of long-term memory, which all require precise regulation of proteome alterations [6]. When the effectiveness of proteostasis systems decreases, the misfolded proteins are deposited within the cell, potentially interrupting regular cellular processes and leading to cell death.

Therefore, identifying the presence of misfolded protein oligomers is essential for detecting disease initiation and progression and developing therapeutics. Several approaches have been proposed to differentiate and quantify protein oligomers’ relative concentrations from their monomeric species. These usually involve the use of fluorescence-based (FRET) [7,8] and other biophysical techniques, like Fourier-transform infrared spectroscopy (FTIR) [8,9], surface plasmon resonance (SPR) [10], and cyclic voltammetry [11,12]. Other techniques, such as circular dichroism (CD), atomic force microscopy (AFM), and transmission electron microscopy (TEM), are equally valuable in revealing structural and morphological differences in protein species. However, they are limited by parameters such as sample purity for AFM, limited resolution for CD, and difficulty in detecting smaller oligomers for TEM [13,14,15]. Real-time quaking-induced conversion (RT-QuIC) and protein misfolding cyclic amplification (PMCA) are highly sensitive protein amplification assays for quantifying misfolded proteins in NDs [16,17]. However, while these approaches have demonstrated high sensitivity and specificity for synucleinopathies [17], they faced challenges when applied to variant CJD and some familial forms of PD/DLB [18,19].

Thioflavin T (ThT) is often used to monitor the aggregation kinetics of amyloidosis in vitro. Still, its specificity is limited to fibrillization since ThT can bind amyloid proteins in the fibril state, such as plaques and tangles, by Aβ and tau, respectively. Thus, other optical-based strategies have been developed to overcome this problem, such as the development of a coumarin–quinolone (CQ) fluorescent probe, which has a higher binding affinity to Aβ with good selectivity against tau, α-synuclein, and islet amyloid polypeptide (IAPP) [20]. Several other strategies used fluorescent probe-quencher systems that competitively bind to Aβ oligomers. Depending on how they were designed, when the probe and quencher are in their free state and are in close proximity, the release of detectable fluorescence is blocked. However, the probe may cross-react with oligomers by binding to them with higher affinity, and the dissociated quencher allows for the release of a false fluorescent signal [21,22,23]. Biophysical techniques produce different spectra depending on the protein species in the sample. An SPR-based biosensor was used to differentiate Aβ oligomers (OAβ) from fibrils by incorporating OAβ and fibril-specific antibodies and immobilizing them on carboxymethylated (CM)-dextran-covered chips through amine coupling [24]. The antibodies successfully captured their respective targets and produced a significant SPR signal. The addition of curcumin also showed inhibition of Aβ aggregation, which was reflected in the decreased SPR signal [24]. FTIR spectroscopy was also used to discriminate AD patients according to the severity of the disease [25]. Despite their exceptional resolution, these methods are laborious and require bulky and expensive instruments. Therefore, diagnostic methods that involve easier handling and implementation are necessary.

## 2. Oligomer Formation and Fibrillization

Accumulations of protein aggregates are a common observed characteristic in various NDs. The aggregates are typically made up of fibrils that contain misfolded proteins exhibiting a β-sheet structure, which are referred to as amyloids. Besides insoluble amyloid fibrils, protein aggregations could also form soluble oligomers and were observed to be involved in multiple processes, such as synaptic dysfunction, reduced long-term potentiation, and induced microgliosis [26]. There is growing knowledge of the active pathways that drive protein aggregation, along with new insights into the molecular causes of cellular damage. These methods would hint at rational therapeutic strategies. Khan et al. have published a comprehensive review that covers the techniques, folding mechanisms, and biochemical properties involved in these diseases [27].

Oligomers, which are an essential step in the fibrillization of proteins by acting as intermediates in amyloid formation, are increasingly recognized as major contributors to NDs [28,29]. Studies have shown that proteins in the oligomeric phase are generally more toxic than mature fibrils [30,31]. Oligomers were also shown to induce apoptosis and inhibit long-term potentiation through cell culture experiments, while fibrils mainly trigger secondary necrosis [32]. Animal studies indicated that α-synuclein variants that enhance oligomer formation cause more significant dopaminergic loss compared to those that form fibrils, highlighting the detrimental effects of oligomers [33].

Oligomers have also been revealed to have varied kinetic and thermodynamic stabilities but have common characteristics: they are mainly non-fibrillar and tend to dissociate back into monomers rather than maturing into fibrils [34]. Non-specific interactions primarily influence oligomer formation, particularly in physiologically relevant concentrations [35]. Oligomerization typically has two phases: hydrophobic coalescence followed by β-sheet-driven reorganization, which potentially exposes hydrophobic regions. These regions are currently hypothesized to contribute to toxicity. In contrast, hydrophobic regions are not as markedly exposed in fibrils [36].

Conformational changes have also been found to be involved in the conversion of Aβ oligomers into fibrils, leading to reduced neurotoxicity in AD. In one study, Aβ oligomers were observed to be initially composed of loose aggregated strands whose C-termini were protected from solvent exchange and which have a turn conformation [37]. During fibrillization, individual β-strands polymerized in a parallel, in-register orientation to form β-sheets [37]. Water has also been shown to be an essential component since it can either accelerate or slow down the process depending on the protein’s hydrophobicity [38]. Lately, a growing number of studies have shown that amyloid oligomers can act as on-pathway precursors or off-pathway competitors of fibrils, and environmental conditions largely determine the type of pathway [39]. This potentially increases the utility of our approach as it can further our understanding of the pathological involvement of oligomers, particularly in diseases where on-pathway oligomers are involved.

## 3. Multimer Detection System

**Figure 1:**
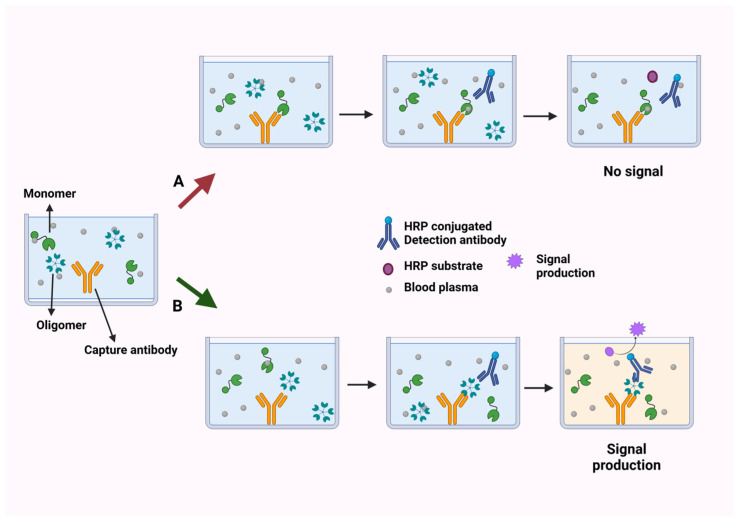
Principle of MDS. Capture and detection antibodies recognize an overlapping sequence of the same epitope. (A) Monomeric forms of the analyte are not detected since the capture antibody already conceals its epitope. (B) Oligomeric forms have other epitopes exposed from clustered monomers, thus allowing the binding of detection antibodies and resulting in signal production. Created with BioRender.com.

The Multimer Detection System (MDS) was initially developed to detect prion oligomers in the blood samples of PrP diseases [40]. The working principle of MDS is simple and foregoes the need for lengthy and laborious steps in other approaches. MDS is an atypical sandwich enzyme-linked immunosorbent assay (ELISA), where the capture and detection antibodies would recognize slightly different but overlapping sections of the same epitope of the target protein (Figure 1). The monomeric form of the analyte would have its epitope covered by the capture antibody, thus preventing the additional binding of the detection antibodies, and no signal would be produced. In the case of oligomers, clustered monomers would have exposed multiple epitopes for the attachment of both capturing and detection antibodies, resulting in a detectable signal despite capture antibodies already having concealed the epitope of one monomer subunit. Therefore, it would be vital to identify a unique exposed epitope of the analyte in the periphery of the oligomer and ensure that enough space is present to permit the proper docking of detection antibodies. For some antibodies where the manufacturer did not describe the immunogen, epitope mapping would be required to identify the region and verify whether it would be exposed to the oligomeric state of the protein [41,42]. When amyloid beta aggregates, the MDS signal increases during the initial phase, where soluble forms of aggregates are generated. This signal decreases in the later phase as insoluble fibrils become more dominant [43]. These findings suggest that MDS may primarily detect soluble forms of aggregates. Given that soluble aggregates are widely recognized as the main toxic species in diseases, this characteristic of MDS could be seen as an advantage. While the ability of MDS to detect a broad range of multimers makes precise quantification difficult, relative concentrations could be measured using artificially generated oligomer-mimicking standard protein (OMSP) of Aβ peptides, allowing it to be applied for diagnostic purposes.

## 4. Expansion of MDS

**Figure 2:**
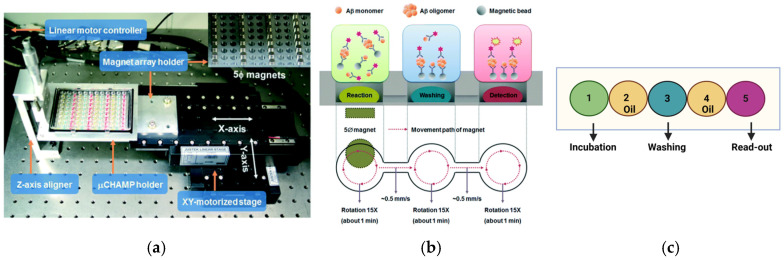
An XY-motorized magnet array stage controls the movement of magnetic beads. (**a**) How the PDMS-μCHAMP appears when connected to the motorized array stage. Permanent neodymium magnets (green bar) are incorporated into a duralumin array holder. Stage movement is controlled by PMAC software, version 1-3, which accounts for linear and circular motion, turn radius, and number of rotations. (**b**) The movement path is described in the layout for MDS. (**c**) Chamber (1) is for incubating antigens and capturing antibody-conjugated magnetic beads and HRP-conjugated antibodies (green reaction solution). Chambers (2,4) contain mineral oil for separating reagents. Chamber (3) is for washing. Chamber (5) is for detecting the signal and includes the HRP-substrate (red reaction solution). Monomers do not bind capture and detection antibodies. Still, oligomer forms have two or more epitopes for antibodies, and they react with the HRP substrate, generating a fluorescence signal [44].

MDS had throughput limitations due to painstaking washes in the ELISA steps, requiring large reagent and sample volumes. To overcome the limitations, improvements were made in MDS by using a droplet-based magnetic bead immunoassay platform [44,45]. The microchannel-connected multi-well plates (µCHAMPs) were constructed from poly-dimethylsiloxane (PDMS) and had the same footprint as a standard 96-well microplate. The difference is that the 96 microwells are arranged into 32 sets of 3, where the three adjacent microwells are connected at the bottom by a microchannel measuring 200–300 µm in height and 0.5–1 mm in width that contains mineral oil [44]. In each set, one microwell contained the reaction droplet, the next contained the washing buffer, and the last included the detection solution that gave off the signal once oligomers were detected (Figure 2). Capture monoclonal antibodies were then conjugated to magnetic beads (2.8 µm) and resuspended in the reaction droplet with recombinant Aβ_42_ that was preincubated at room temperature for three days to form OAβs. The reaction solution also contained previously prepared OMSP of Aβ_15_ peptides and horseradish peroxidase (HRP)-conjugated monoclonal antibodies. A motorized stage capable of linear and circular movement holding neodymium magnets was placed below the µCHAMP (Figure 2a). The linear movement carried the magnetic beads across different solutions through the microchannels that connected adjacent microwells. The circular movement allowed efficient mixing of the magnetic beads as they passed to the next droplet (Figure 2b). Any bound HRP-antibodies on the microbeads that reached the detection droplet in the third microwell due to Aβ oligomer’s presence resulted in oxidation of the substrate and an observable signal. The motorized nature of this approach and its ability to perform several reactions in parallel significantly increase the throughput of MDS. This method’s detection limit for soluble OAβ is ~3 pg/mL. The microliter-sized volumes of reagents and samples used also contributed to its cost-effectiveness.

In the microchip-based method employing micropillars, the chip compartments are filled with water-based chemicals or oil for sequential testing processes, with micro-pillar patterns between chambers to create reinforced barriers between water and oil (Figure 2c) [45]. Using these tiny columns, magnetic beads could successfully move through each compartment by moving a magnet in a straight line across the microchip. The fluorescence intensity proportionally increased based on the serially diluted Aβ standard solution concentrations. The results from the test showed that the detection limit was approximately 10 pg/mL despite being carried out using manual magnet manipulation. This platform made the intricate ELISA procedure easier and achieved a sensitivity comparable to that of the conventional magnetic bead immunoassay. Overall, the magnetic bead-droplet system decreased the testing time and the amount of antibody required for diagnosis.

## 5. Applications of MDS in NDs

### 5.1. Prion Disease

Prion disease or transmissible spongiform encephalopathy (TSE) is a zoonotic fatal ND referred to as scrapie in sheep and goats, bovine spongiform encephalopathy in cattle, and Creutzfeldt–Jakob disease (CJD) in humans [46]. TSE diseases are characterized by spongiform brain lesions with nerve damage and deposition of abnormal aggregation of infectious prion protein (PrP^Sc^) in the central nervous system (CNS) [47]. PrP^Sc^, a putative transmissible agent, has the property of converting additional normal prion (PrP) to become pathogenic and is associated with infectious diseases resulting from the consumption of PrP^Sc^-infected meat in variant CJD and administrations of growth hormone or medical devices in iatrogenic CJD [47].

PMCA [48] and RT-QuIC analysis [49] are representative amplification technologies for diagnosing PrP diseases that can detect low levels of PrP^Sc^ in the cerebrospinal fluid (CSF) through the addition of a PrP substrate and breaking the fibrils by shaking or sonication. However, these techniques cannot discriminate molecular subtypes of CJD and are time-consuming, making them unsuitable for high-throughput diagnostic tests in medical facilities [50]. Meanwhile, MDS is a fast yet accurate TSE screening technique that provides results within hours without needing protein amplification. MDS was initially developed to detect PrP^Sc^ oligomers in the blood of scrapie-infected animals specifically, and anti-PrP monoclonal antibodies (3O8 and 3F4) with overlapping epitopes were used for detection [40]. The oligomeric PrP was successfully detected in plasma samples of prion-infected hamsters. In a similar study, PrP^Sc^ was detected in the plasma of scrapie-infected sheep with 100% sensitivity and specificity [42]. In a blind-validation study, diseased sheep and healthy controls were successfully identified with a sensitivity of 92% and a specificity of 100% [42]. The improvised MDS system with a boron-doped diamond (BDD) electrode detected PrP^Sc^-infected sheep’s plasma through electrochemical means using magnetic microparticle multimers. The magnetic microparticles were not in direct contact with the BDD electrode, which prevented fouling and enabled quick electrochemical detection with minimal sample volumes. The key benefits of the electrochemical fluidic system included recyclability and sensitivity. Additionally, the electrochemical testing findings aligned with chemiluminescence testing in the same study [51].

The application of MDS in PrP diseases could monitor potential drug efficacy through screening inhibitors for the oligomerization of PrP, and it may be helpful in early diagnosis before extensive damage from PrP^Sc^ begins.

### 5.2. Alzheimer’s Disease (AD)

It was evident from multiple studies that OAβ is the most harmful Aβ species in AD. It is responsible for synaptic dysfunction, tau pathology, neuroinflammation, interrupted axonal transport, and neuronal death [52]. Additionally, OAβ is more closely associated with cognitive symptoms than Aβ plaque counts are [53], suggesting that OAβ could better represent clinical symptoms and AD progression than Aβ plaque burden. Although specific studies involved labor-intensive and time-consuming techniques for detecting OAβ, like immunoprecipitation mass spectrometry (IP-MS), single-molecule array (Simoa) assays, and neuroimaging [54], MDS streamlined the high-throughput analysis. The MDS technique resembles an ELISA in terms of simplicity and potential for automation, making it suitable for broad implementation. By adapting it accordingly, MDS can selectively detect OAβ by having two different antibodies competing for the same region at the N-terminus of Aβ [55]. The OAβ test’s effectiveness in detecting AD patients is improved by exposing plasma samples to artificial Aβ_42_ before conducting the test [43].

MDS evaluated the oligomerization tendency of Aβ and corresponded to the sigmoid function of Aβ accumulation [56] with a sensitivity of 83.3% and specificity of 90% [43,57]. With increasing age, a higher tendency for plasma OAβ levels was detected by MDS [58]. In a tester-blinded study using a protocol approved by the Ministry of Food and Drug Safety (MFDS), MDS of OAβ (MDS-OAβ) was able to distinguish between AD dementia (ADD) and a group of cognitively normal individuals with a sensitivity of 100% and specificity of 92% [56]. It displayed a strong ability to predict central amyloidopathy and clinical AD with high accuracy [59,60]. The studies found elevated levels of oligomers in individuals with mild cognitive impairment (MCI) or the early stages of AD compared to those in the advanced stages [61]. This validated the theory that amyloidopathy advances in the initial phase of AD and stabilizes in the later stage [62]. MDS-OAβ could forecast cognitive decline over time in subjective cognitive decline (SCD) and MCI patients, as assessed by subsequent mini-mental state examination (MMSE) [63]. Moreover, the validity of MDS was evaluated by examining the correlation between OAβ in the plasma and traditional Aβ biomarker (Aβ_42_, phosphorylated-tau, total tau) levels in CSF, amyloid positron emission tomography (PET), and brain-volume reductions detected using three-dimensional T1 magnetic resonance imaging (MRI) [43,59,60]. In a study using plasma MDS-OAβ, a sensitivity of 76% and specificity of 67% were observed for predicting amyloid PET positivity, and this technique reduced the costs and number of PET scans needed to screen for amyloidosis [64]. Additionally, the area under the curve (AUC) of plasma OAβ was 0.844, showing no significant difference compared to conventional AD biomarkers (*p* = 0.250) [55]. Thus, measuring OAβ levels through MDS can reflect AD pathology and help diagnose AD. MDS-OAβ has been authorized for use in clinical settings after receiving approval from the MFDS and the National Evidence-based Healthcare Collaborating Agency (NECA) in Korea [65] and is patented by People Bio Inc., Korea [66]. The commercial kit developed by this company effectively distinguished between AD patients and cognitively normal individuals with 100% sensitivity and 92% specificity, using a sample size of fifty [56]. A systematic interpretation of MDS-OAβ, neuropsychological test, and brain MRI (Table 1) could support accurate diagnosis and staging of AD [65].

Some EOAD cases highlight the importance of early detection through diagnostic tools like MDS. A Korean male with the *PSEN1* His214Asn mutations developed EOAD at 41, with positive amyloid-PET scans and increased OAβ detected in his blood [67]. Similarly, a French patient in Korea with the *PSEN1* Glu318Gly mutation and additional genetic risk factors exhibited memory decline in his early 60s, with elevated OAβ supporting the AD diagnosis [68]. Another case involved a Korean woman with a novel *APP* Val669Leu mutation, who experienced rapid cognitive decline at 56, with significantly elevated OAβ detected in plasma [69]. These cases underscore the valuable role of early detection using MDS to identify elevated protein oligomers, which can indicate the onset of AD even before the manifestation of severe symptoms, potentially allowing for earlier intervention and improved disease management.

In some studies, MDS-OAβ levels were calculated as ratios of the oligomeric concentrations measured in patient groups compared to those in control groups to illustrate their relationship and differences better. MDS-OAβ ratios greater than 1 in individuals with normal cognitive impairment (NCI) or subjective cognitive impairment (SCI) may indicate the early stage of AD. Alongside AD progression, there is a gradual decrease in the expression levels of Aβ [56]; thus, a low OAβ level (<0.5) indicates late-stage AD. Furthermore, reduced Aβ levels in individuals with AD may also indicate limbic-predominant age-related TDP-43 encephalopathy (LATE). Finally, individuals with dementia were observed to have minimal amounts of OAβ. An MDS-OAβ ratio higher than 1 in these individuals may suggest the presence of AD subtypes or a combination of different pathologies [70].

Elevated plasma MDS-OAβ levels displayed a significant negative correlation with MMSE (r = −0.29, *p* < 0.01) and CSF Aβ_42_ (r = −0.20, *p* < 0.05) and a positive correlation with CSF tau (r = 0.20, *p* = 0.01) [64]. A similar correlation was observed in another study with MMSE (r = −0.43, *p* = 0.02), cognitive abilities screening instrument (CASI) (r = −0.56, *p* < 0.01), catechol-O-methyltransferase (COMT) (r = −0.45, *p* = 0.01 for immediate recall; r = −0.56, *p* < 0.01 for 5-min delayed recall; r = −0.71, *p* < 0.001 for 10-min delayed recall), and Alzheimer’s disease assessment scale-cognitive (ADAS-cog) scores (r = 0.59, *p* < 0.001) [56]. Plasma MDS-OAβ, along with apolipoprotein E (APOEε4) genotype and age, effectively detected brain amyloidosis in a large Aβ-confirmed group [64]. In a recent finding, MDS-OAβ was negatively correlated with cognitive function and the thickness of the left fusiform gyrus. In addition, MDS-OAβ showed a direct correlation with cerebral Aβ accumulation in all stages of AD [71]. Plasma OAβ levels also negatively correlated with the MRI results, which showed a reduction in brain volume. A negative correlation of increased OAβ with reduced volume in the bilateral temporal, amygdala, parahippocampal, lower parietal lobe, left cingulate, precuneus regions, left temporal lobe, and posterior corpus callosum was also reported [60]. It suggested that MDS-OAβ is linked to Aβ levels in the brain besides cognitive status in different stages of AD. Despite the significant correlation, more longitudinal studies should be conducted to reinforce MDS as a valid AD diagnostic assay. Combining plasma Aβ, tau, and other assays will probably improve AD diagnosis and potentially reduce the need for more invasive and expensive techniques. Plasma exposed to various anticoagulants would utilize distinct mechanisms to measure the levels of MDS-OAβ. The studies using heparin-based plasma have been more thoroughly validated [56] than ethylenediaminetetraacetic acid (EDTA)-based ones. Given the ease of obtaining blood samples, EDTA-based MDS-OAβ could serve as an alternative for screening. A comparison between heparin-based MDS-OAβ and EDTA-based MDS-OAβ is necessary to determine their effectiveness in prescreening for amyloid PET positivity. EDTA-based MDS-OAβ machine learning models can also be used to identify AD risk in patients [72].

Tau is a microtubule-associated protein that regulates microtubule assembly and structural stability in neurons [73]. Tau undergoes oligomerization under physiological conditions to become dimers and trimers, and these species are distinct from pathological aggregates [74,75]. The N-terminal region of tau mediates oligomerization [76], while the interaction with heat shock protein 90 (Hsp90) promotes an open tau conformation, facilitating oligomer formation [77]. Stable tau oligomers can be released and internalized by cells and do not necessarily lead to tau aggregation [78]. Pathologically speaking, tau hyperphosphorylation is the main driver of abnormal aggregation and stems from an imbalance in kinase and phosphatase activity [79].

Tau proteins usually unfold but can form filaments in NDs through self-assembly and stacking. In AD, tau filaments consist of parallel, in-register β-strands that stack on each other, with existing filaments serving as templates for incoming tau molecules [80]. This stacking is modulated by amino acid side chains, particularly arginine, which interact through π-stacking [81]. The core region of tau filaments adopts a cross-β structure, while the rest remains primarily unfolded [82]. Interestingly, tau can form disease-specific conformations, as seen in distinct folds observed in Pick’s disease compared to AD [83]. These structural differences may explain the varying pathological presentations in different tauopathies [84,85].

Recent research highlights the potential of tau oligomers as diagnostic biomarkers. This need was driven mainly by the critical role of misfolded and accumulated tau in AD and other NDs [86]. For large-scale studies, simple, versatile, and economical assays are required. Blood-based biomarkers, including phosphorylated tau (p-tau), show promise for non-invasive AD monitoring [87]. Though p-tau is a well-established AD biomarker, elevated levels of which in CSF, along with total tau and decreased Aβ_42_, are central to research criteria for AD diagnosis; CSF p-tau levels in some tauopathies like progressive supranuclear palsy (PSP) and Pick’s disease remain unchanged despite observable tau pathology [88]. In contrast, tau oligomers are toxic intermediates in tau aggregation. They may be more directly related to neurotoxicity than early diagnosis, making them valuable for differentiating PSP from other Parkinsonian disorders [89,90]. Therefore, measuring the concentrations of the two tau species may significantly improve diagnostic accuracy.

### 5.3. Frontotemporal Dementia (FTD)

FTD is characterized by progressive atrophy of the frontal and anterior temporal lobes [91,92]. It ranked third among the most common causes of early-onset dementia (EOD), usually presenting between ages 45 and 65 [93]. The main variants of FTD include the behavioral variant FTD (bvFTD), semantic dementia (SD), and progressive non-fluent aphasia (PNFA) [94].

Neurofilament light chain (NfL) has emerged as a significant diagnostic and prognostic marker with elevated levels in CSF and blood of FTD compared to healthy controls and other dementias [95,96,97,98,99]. However, NfL is limited in distinguishing slow progressors from phenocopies and lacks the specificity for FTD [96,97]. Tau also plays a key role in FTD pathology, especially in PSP and corticobasal degeneration, where tau aggregation predominates. Elevated pathological tau oligomers, detectable by MDS, serve as critical biomarkers in these diseases. In contrast, TDP-43 is a compelling biomarker implicated in most of the cases involving SD, where it plays a vital role in the underlying pathology [100,101]. The ability of MDS to quantify both tau and TDP-43 pathological oligomers may provide additional insight into the molecular mechanisms underlying different FTD variants. In a recent study, MDS showed increased concentrations of oligomeric TDP-43 (o-TDP-43) in the plasma of Korean FTD patients with SD compared to those in patients with MCI, early-onset AD (EOAD), late-onset AD (LOAD), idiopathic PD (IPD), and healthy controls [102]. Moreover, the ratio of o-TDP-43 to total TDP-43 (t-TDP-43) showed an improved AUC value (0.916) than o-TDP-43 concentrations alone (0.904) [102]. FTD represented a complex spectrum of disorders, and only SD was consistently associated with TDP-43 positive pathology [103]. Given the complexity of dementias involving TDP-43 proteinopathy, a new scheme was developed to determine the likelihood of a patient having LATE with neuropathological changes (LATE-NC), a form of dementia triggered by TDP-43 proteinopathy, in comparison to a patient with AD with neuropathological changes (ADNC) [104]. Despite this, the scientists claimed that current methods of biomarker analysis could only rule out amyloidosis [104]. Applying MDS to o-TDP-43 could present a promising approach to enhance our understanding of the molecular mechanisms underlying TDP-43 pathology. MDS would provide additional data that may support the development and refinement of diagnostic criteria for NDs involving TDP-43. However, this exploratory study incorporated a limited number of SD patients (*n* = 16), and further extensive studies are warranted to verify this correlation [102].

### 5.4. Synucleinopathies and Tauopathies

Alpha-synuclein and tau oligomerization are implicated in several NDs. PD and DLB involve α-synuclein oligomers, which are toxic and worsen neurodegeneration [105,106]. Tau oligomers are associated with AD and other tauopathies [107]. MDS may be a useful approach to quantify the concentrations of these oligomers in plasma and other biofluids when designed with overlapping antibodies that recognize the proteins of interest. Interestingly, α-synuclein and tau can form hybrid oligomers in synucleinopathies, suggesting a potential interaction between these proteins [108,109]. By assuming that the capture and detecting antibodies bind to epitopes that are not in the interaction site that created the hybrid oligomer, MDS can also quantify these hybrid oligomers to help understand the progression of multiple proteinopathies. MDS also has the potential to be further developed into a panel that could differentiate NDs. Different protein oligomers, the diseases they are associated with, and the respective diagnostic applicability of MDS are summarized (Table 2).

### 5.5. Huntington’s Disease

Huntington’s disease (HD) is caused by a CAG repeat expansion in the huntingtin (*HTT*) gene, resulting in an elongated polyglutamine tract in the protein [110]. Recent research has revealed that mutant huntingtin (mHtt) aggregation involves liquid-liquid phase separation, potentially contributing to HD pathogenesis [111,112]. The subcellular localization and properties of these aggregates correlate with symptom onset and progression [113]. Importantly, HD affects neurodevelopment, disrupting neural progenitor cell polarity and differentiation [114]. Various therapeutic strategies targeting mHtt are being explored, including antisense oligonucleotides and RNAi approaches [115]. However, caution is needed since wild-type Htt (wt-Htt) plays a critical role in synaptic function. Its loss may have detrimental effects [116]. Since the N-terminal region of Htt contains the expanded polyglutamine domain and is separated from the full-length protein through proteolytic processing and aberrant splicing [117], exposed epitopes in the N-terminal region may theoretically be mapped to develop an MDS targeting oligomerized Htt.

Various factors influence Htt oligomerization. Oxidation of cysteine residues within the N-terminal region promotes oligomer formation and delays the clearance of soluble mutant Htt [118]. The first 17 amino acids in the N-terminus of Htt play a crucial role in initiating oligomerization by facilitating the formation of α-helical intermediates [119,120,121]. Oligomers represent the most active lipid-binding species, with structural flexibility being key to membrane interaction [120,122]. The polyglutamine (poly-Q) tract length directly correlates with oligomer formation propensity and HD severity [123]. Interestingly, mHtt can seed oligomers involving wt-Htt, suggesting a prion-like spreading mechanism [124]. Visualization techniques have been developed to study Htt oligomerization and cell-to-cell transmission [125].

During Htt oligomer stacking, the 17 N-terminal amino acids play a crucial role in forming α-helix-rich oligomeric intermediates, which serve as precursors for amyloid formation [126]. These oligomers are structurally flexible, enhancing their ability to bind lipid membranes [120]. The expanded poly-Q segment also accelerates oligomer formation and subsequent nucleation [126]. Full-length Htt exon-1 peptides form spherical oligomers containing 100–600 molecules, which then assemble into protofibrils [127]. The aggregation process involves competing pathways, with the Htt NT-mediated pathway dominating under normal conditions [126]. Interestingly, monomers were not observed in cell culture expressing expanded poly-Q Htt exon-1, with tetramers being the most minor detectable form [127].

## 6. Future Applications: Hybrid Oligomers

More research suggests that hybrid oligomers, composed of multiple misfolded proteins, can form in vivo in NDs. Studies have demonstrated the co-occurrence and interaction of Aβ, α-synuclein, tau, and other proteins in oligomeric assemblies in AD and PD [108,128,129]. These hybrid oligomers may contribute to disease pathogenesis by altering neuronal activity and promoting cross-seeding of protein aggregation [130].

Recent research has revealed that TDP-43 interacts with annexin A11 (ANXA11) to form heteromeric amyloid filaments in frontotemporal lobar degeneration with TDP-43 inclusions (FTLD-TDP) Type C [131]. This interaction involves the low-complexity domains (LCDs) of both proteins, forming an extensive hydrophobic surface [131]. ANXA11 aggregates colocalize with TDP-43 inclusions in various neurodegenerative conditions, including ALS and FTLD-TDP [132]. TDP-43 has been shown to interact with numerous proteins involved in RNA metabolism, stress granule formation, and vesicular trafficking [133,134]. Mutations in both TDP-43 and ANXA11 have been associated with ALS and FTLD, underscoring their importance in disease pathogenesis [134,135]. The formation of oligomers appears to be an early event in disease progression, with intraneuronal accumulation preceding extracellular deposition [136]. Different classes of oligomers have been identified, with varying spatial distribution and potential for neural dysfunction [137]. To this point, the versatility of MDS is a significant advantage that allows the assay to be specifically designed to recognize hybrid oligomers when their unique exposed epitopes are identified. However, the utilization of MDS on hybrid oligomers is an avenue yet to be explored.

## 7. Gaps and Limitations

Despite the broad applications of MDS, it has several limitations that need to be considered. First, the method can only detect proteins with exposed epitopes. Oligomers with limited exposed epitopes decrease the chances of an antigen–antibody interaction. The unstable nature of the oligomers can potentially hide the target epitope or be masked by the presence of other proteins. In cases where the epitope is composed of a shorter sequence, this decreases interaction specificity and significantly increases the chances of cross-reacting with other proteins [138]. Repetitive sequences are also crucial as they can lead to extensive cross-reactivity and provide an apparent increase in the generated signal [139]. MDS also struggled to detect other proteins, especially those that form disordered oligomers. Our group was unsuccessful in attempting to detect alpha-synuclein oligomers in the plasma of PD patients. We attributed this to the natural tendency of the protein to form tetramers and make it resistant to aggregation [130,140,141]; however, this aspect of the assay warrants further investigation to understand the underlying reasons for its occurrence.

Another challenge faced in developing MDS was its low sensitivity, so a pre-treatment step, which is essentially seeding the samples with synthetic Aβ_42_, was required to amplify the minute oligomer levels; thus, MDS is fundamentally measuring the oligomerization tendency of the sample instead of the actual oligomer concentrations already in the sample [55]. This is important as there may be unknown factors that contribute to amplifying this signal, which are not necessarily exclusively dependent on the initial concentrations of the target protein. This also adds another factor to consider in the assays, which is the uniformity of the seeding concentrations of synthetic Aβ_42_ [58]. Oligomer concentrations are also prone to periodic fluctuations on a daily, sometimes hourly, basis; thus, methods to normalize these intra- and inter-individual variations are required [55]. Moreover, the preparation of the synthetic oligomers also adds complexity to the assay workflow [58]. The additional pre-incubation step, which takes six days [56], dramatically increases the total run-time of the assay.

Several gaps also need to be addressed in understanding the parameters governing MDS. The negative effects of long-term storage of samples on the concentrations of detected MDS-OAβ have been observed and are still unexplained. Storing plasma samples for more than four years resulted in no significant differences between individuals with normal and abnormal amyloid levels [64]. More studies are needed to determine the effects of long-term storage and optimal storage conditions [64]. Additionally, the exact mechanism by which spiked synthetic Aβ induces Aβ oligomerization in AD plasma samples is still not fully understood [56]. Similarly, the actual conformational structure of the multimeric PrP^Sc^ forms detected by MDS is unclear. There is also a possibility that the measurements from healthy controls included protease-resistant PrP^Sc^ [40].

## 8. Conclusions

The diagnostic potential of MDS is very much apparent in AD and prionopathies and consistently showed strong correlations of increased OAβ concentrations with neurodegeneration and increased expression of etiologically related markers. In the future, its scope can still be extended to other proteinopathies unrelated to neurodegeneration, such as amylin in T2D [142], p53 in cancers [143], and mutant transthyretin-causing cardiomyopathy [144]. These diseases also result from the oligomerization of proteins due to structural mutations; therefore, the potential for MDS will be equally worth exploring among them. OAβ detection is restricted to proteins where overlapping epitopes are exposed to the surface for capture and detection by MDS. It would be essential to note the location and orientation of the target immunogen of these oligomers when selecting the antibodies in MDS to ensure their proper binding. Several tests also need to be performed to evaluate the cross-reactivity of the selected antibody, especially when the epitope in consideration lacks complexity. Unpredictability in the folding of oligomers that depend on solution conditions like pH, temperature, and the presence of proteases may also significantly affect the position or integrity of the target immunogen; therefore, these would be additional concerns that need to be addressed when developing MDS. Furthermore, processes like blood drawing, sample preparation, and variability introduced by the individual performing the tasks can affect the results. To ensure consistency, outsourcing to specialized institutions or implementing large-scale equipment is recommended. Finally, the interaction dynamics of the oligomers and antibodies in MDS remain unclear and may be considered an interesting avenue in future research. If we can elucidate and explain how oligomers interact with proteins, we can utilize this knowledge not only for diagnostic capabilities but also for developing therapeutic strategies.

Despite the current gaps and limitations, MDS-OAβ has attained good diagnostic capability (78.3% sensitivity and 86.5% specificity), and its future use to detect a wide range of oligomers can be explored. Additionally, being a minimally invasive method, the blood sample for MDS can easily be obtained in primary care facilities. This simplicity would be beneficial for monitoring disease progression since it could be repeated several times with little impact on the patients. The data obtained from testing biomarkers in NDs are compelling, indicating that this assay is ready to establish its relevance in other unrelated diseases. People (>65 years) with NDs (including hereditary NDs) often had a combination of one or more proteinopathies, which increased with age [145]. For example, AD is commonly linked with aging-related tau astrogliopathy (ARTAG), TDP-43/LATE-NC, DLB, and cerebrovascular disease (CVD). Such mixed pathologies contribute to the intricacy of categorizing NDs and pose challenges for concepts related to disease development, as well as biomarkers and studies using neuroimaging. Therefore, careful investigation by MDS is necessary to distinguish these neurodegenerative proteinopathies.

Early diagnosis is crucial for initiating timely and effective treatment for neurodegenerative diseases. Since oligomers form in the early stages of these diseases, MDS is particularly well-suited for early detection, enabling the implementation of appropriate treatment strategies. While treatment outcomes often guide the future direction of therapy, fibril forms have proven unreliable as biomarkers and current methods rely on measuring total protein concentrations. However, oligomers, as the toxic species, are likely the most accurate indicators for evaluating therapeutic efficacy. By addressing these challenges, MDS offers significant potential to bridge existing gaps and advance both early diagnosis and the monitoring of treatment outcomes in neurodegenerative diseases.

## Figures and Tables

**Table 1:** The probable diagnosis of AD is based on the MDS-Oaβ, a neuropsychological test, and brain MRI [65].

MDS-OAβ	Amyloid PET	MMSE Score	Most Probable Diagnosis
Negative	Abnormal	Normal	Other NDs
	Normal	Normal	Normal
	AD-compatible atrophy	Cognitive impairment	Other NDs
	Normal	Cognitive impairment	Other reasons for cognitive impairment
Positive	AD-compatible atrophy	Normal	Preclinical AD
	Normal	Normal	Preclinical Amyloidopathies
	AD-compatible atrophy	Cognitive impairment	AD
	Normal	Cognitive impairment	An early stage of AD

**Table 2:** Overview of neurodegenerative disease-associated proteins, their associated diseases, and the sample sources used for MDS. The table illustrates how MDS has been applied to different proteins and sample types, highlighting its potential in distinguishing between neurodegenerative diseases.

Protein	Associated Disease	Sample Source Used in MDS	Disease Differentiation Potential	Key Publications
Prion	CJD, Scrapie	Scrapie-infected hamster plasma	Detection of prion protein multimers in plasma of scrapie-infected hamsters	An, 2010 [40]
		Scrapie-infected sheep plasma	MDS detected PrP Sc in plasma samples from scrapie-infected sheep with clinical samples with 100% accuracy.	Lim, 2015 [42]
A-beta	AD, LATE-NC, DLB	AD plasma	Differentiated AD patients from healthy controls with 78.3% sensitivity and 86.5% specificity	Lee, 2020 [55]
		AD plasma (heparin-treated)	Differentiated AD patients from normal controls with 100% sensitivity and 92.31% specificity using a cutoff of 0.78 ng/mL	Youn, 2020 [56]
		Abnormal amyloid status in plasma	Identification of individuals with abnormal amyloid status with an AUC of 0.74, which improved to 0.81 when combined with APOE4 and age	Mofrad, 2021 [64]
		AD plasma	Differentiated dementias due to AD versus non-AD etiologies	Dominguez, 2022 [70]
TDP-43	AD, LATE, DLB, FTD	FTD plasma (semantic dementia)	The ratio of o-TDP-43 to t-TDP-43 concentrations in semantic dementia	Jamerlan, 2023 [102]
A-syn	LATE-NC, PD, DLB	No data yet		
Tau	CJD, AD, LATE-NC, DLB, PNFA, bvFTD	No data yet		
Huntingtin	HD	No data yet		
Hybrid	PNFA, bvFTD	No data yet		

## Data Availability

Not applicable.
